# Identification of early cardiac dysfunction and heterogeneity after pressure and volume overload in mice by high-frequency echocardiographic strain imaging

**DOI:** 10.3389/fcvm.2022.1071249

**Published:** 2023-01-13

**Authors:** Ran Xu, Zhiwen Ding, Hao Li, Jing Shi, Leilei Cheng, Huixiong Xu, Jian Wu, Yunzeng Zou

**Affiliations:** ^1^Shanghai Institute of Cardiovascular Diseases, Zhongshan Hospital and Institutes of Biomedical Sciences, Fudan University, Shanghai, China; ^2^State Key Laboratory of Cardiovascular Disease, Fuwai Hospital, National Center for Cardiovascular Diseases, Chinese Academy of Medical Sciences and Peking Union Medical College, Beijing, China; ^3^Department of Echocardiography, Zhongshan Hospital, Fudan University, Shanghai, China; ^4^Department of Ultrasound, Zhongshan Hospital, Fudan University, Shanghai, China

**Keywords:** strain imaging, transverse aortic constriction, aortic regurgitation, left ventricle, pressure overload, volume overload

## Abstract

**Object:**

Aortic stenosis and regurgitation are clinically important conditions characterized with different hypertrophic types induced by pressure or volume overload, respectively, but with comparable cardiac function in compensated stage. Speckle-tracking based strain imaging has been applied to assess subtle alterations in cardiac abnormality, but its application in differentiating these two types of ventricular hypertrophy is still sparse. Here, we performed strain imaging analysis of cardiac remodeling in these two loading conditions.

**Methods:**

C57BL/6J mice were subjected to transverse aortic constriction (TAC)-induced pressure overload or aortic regurgitation (AR)-induced volume overload. Conventional echocardiography and strain imaging were comprehensively assessed to detect stimulus-specific alterations in TAC and AR hearts.

**Results:**

Conventional echocardiography did not detect significant changes in left ventricular systolic (ejection fraction and fractional shortening) and diastolic (E/E’) function in either TAC or AR mice. On the contrary, global strain analysis revealed global longitudinal strain and strain rate were remarkably impaired in TAC while preserved in AR mice, although global radial, and circumferential strain and strain rate were significantly reduced in both models. Regional strain analysis in the long axis demonstrated that longitudinal strain and strain rate in all or most segments were decreased in TAC but maintained or slightly dented in AR mice, while radial strain and strain rate indicated overt decline in both models. Moreover, decreased radial and circumferential strain and strain rate were observed in most segments of TAC and AR mice in the short axis.

**Conclusion:**

Strain imaging is superior to conventional echocardiography to detect subtle changes in myocardial deformation, with longitudinal strain and strain rate indicating distinct functional changes in pressure versus volume overload myocardial hypertrophy, making it potentially an advanced approach for early detection and differential diagnosis of cardiac dysfunction.

## 1. Introduction

Heart failure is a leading cause of morbidity and mortality worldwide, with cardiac hypertrophy as its independent risk factor (1, 2). Pressure overload induced by hypertension and aortic stenosis and volume overload induced by valve regurgitation are clinically important pathogenesis to induce cardiac hypertrophy that are increasingly common in the aging world population. Although both pressure overload and volume overload are characterized with increased mechanical overload, they present distinct hypertrophy types, in which pressure overload causes concentric hypertrophy while volume overload induces eccentric hypertrophy. It is recently found that regression of heart failure is associated with early normalization of ventricular hypertrophy that precedes restoration of cardiac function (2). It is therefore especially important to monitor left ventricular (LV) function to determine the efficacy of therapies.

Conventional echocardiography is widely used to evaluate cardiac function and the severity of heart damage due to its non-invasive and easily available feature. However, conventional LV function indexes such as ejection fraction (LVEF) and fractional shortening (LVFS) are not able to discriminate between the two types of LV hypertrophy (3, 4). Our recent study demonstrates that in compensated phase, LVEF and LVFS are preserved in mice with pressure overload and volume overload (4). Thus, there is an urgent need to identify early functional deficiency and further undertake comparative analysis in LV hypertrophy under pressure and volume overload.

Echocardiographic speckle-tracking based strain imaging, also known as deformation imaging, is a technological advancement that has been developed to objectively quantify global and regional myocardial function (5, 6). Strain imaging has emerged as a promising means for the evaluation of myocardial function both in humans and animal models, being superior to the conventional echocardiographic measurements (7–10). Previous studies have shown that the indexes derived from strain imaging are consistent with the parameters of cardiac magnetic resonance in assessing cardiac volume and ejection fraction (11, 12). In addition, there is a good correlation between strain imaging derived-metrics and invasive cardiac hemodynamic measurements in the evaluation of heart function (13, 14). With the advancement of high frequency ultrasound, the strain imaging has been applied to murine models of myocardial infarction (15), pressure overload (16), and aging (10). However, few studies have ever evaluated the strain imaging of ventricular hypertrophy under volume overload, in comparison with that under pressure overload. In this study, we aim to utilize the speckle-tracking based strain imaging to evaluate cardiac dysfunction in mouse models of pressure overload and volume overload, and to elaborate the differences in LV myocardial activities between these two pathophysiological conditions.

## 2. Materials and methods

### 2.1. Animal models

The animal study protocol was approved by Animal Care and Use Committee of Zhongshan Hospital, Fudan University and were in accordance with National Institutes of Health Guide for the Care and Use of Laboratory Animals (No. 85-23, revised 1996, Bethesda, MD, USA). Adult C57BL/6J male mice (10–12 weeks old) were obtained from the Shanghai Branch of the National Rodent Laboratory Animal Resources (Shanghai, China). Animals were fed at 24 ± 2°C with 12 h-light/12 h-dark cycles. We used transverse aortic constriction (TAC) model and aortic regurgitation (AR) model to induce pressure overload and volume overload, respectively, as we described previously (4). Mice were randomly assigned into four groups: Sham-T group (sham TAC operation, *n* = 8), TAC group (*n* = 12), Sham-A group (sham AR operation, *n* = 8), and AR group (*n* = 12). Briefly, surgeries were performed under mixed anesthesia (i.p. 150 mg/kg ketamine and 10 mg/kg xylazine). For TAC model, 27-gauge needle were placed on the aorta between the innominate artery and the left common carotid artery. After ligated with 6–0 silk, the needle was removed to generate aortic constriction. The Sham-T group underwent a similar surgical procedure but without the ligation. For AR model, a flexible catheter containing a metal wire was inserted into the right common carotid artery and advanced to the aortic orifice under the guidance of ultrasound imaging, and then the metal wire was pushed to puncture the aortic valvular cusp repeatedly. When Doppler ultrasound showed diastolic flow reversal [around 400 mm/sec, we chose this peak velocity to ensure the mean LV wall stress was similar in both TAC and AR mice (4, 17)] at the aortic arch, the catheter with wire were withdrawn. The Sham-A group was performed the same procedure but without puncturing the aortic valve. We chose two weeks post-surgery as the end point of the observation, because our preliminary study showed that the compensated cardiac hypertrophy was observed 2 weeks after surgeries of TAC and AR, with LVEF and LVFS comparable between the mice with pressure/volume overload and the sham-operated controls (4).

### 2.2. Conventional echocardiography

**FIGURE 1:**
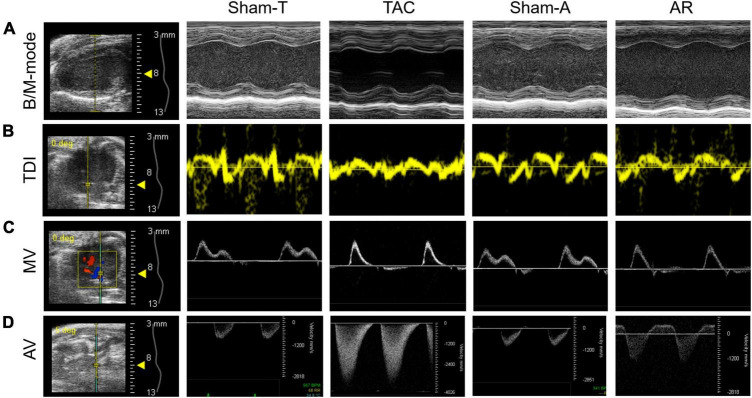
Representative images of conventional echocardiography. **(A)** Mouse B-mode and M-mode images of left ventricle in long-axis view. **(B,C)** Tissue Doppler imaging (TDI) and mitral valve (MV) Doppler was performed to record mitral annular velocity and mitral inflow curves. **(D)** Aortic valve (AV) Doppler was conducted to generate aortic arch flow in systole and diastole. Sham-T, sham TAC operation; TAC, transverse aortic constriction; Sham-A, sham AR operation; AR, aortic regurgitation.

Transthoracic echocardiography was performed 2 weeks after surgery. Briefly, mice were placed on a heating pad to maintain temperature at 37°C and limb electrodes were used to record the electrocardiogram. Mice were anesthetized with 1.5% isoflurane to keep the heart rate (HR) above 450 beats per minute during the procedure. Echocardiographic parameters were acquired from the Vevo 2100 system (VisualSonics, Toronto, Canada) with a 30 MHz transducer. B-model and M-mode images were obtained in parasternal long-axis view and mid-papillary level short-axis view. Then conventional echocardiographic parameters were measured or calculated as follows: LVEF, LVFS, LV internal dimension in systole and diastole (LVIDs and LVIDd), LV posterior wall thickness in systole and diastole (LVPWs and LVPWd), LV end-systolic and end-diastolic volume (LVESV and LVEDV) (Table 1). Moreover, pulsed wave Doppler was performed at the tip of the mitral leaflet in the apical four-chamber view. By simultaneously recording the mitral inflow curves, peak velocity of the early ventricular filling wave (E wave) and tissue Doppler mitral annular velocity, ratio of E wave to E’ wave (E/E’), isovolumic relaxation time (IVRT), isovolumic contraction time (IVCT), and ejection time (ET) were evaluated (Figure 1). Considering merged mitral E and A waves were found in most cases of AR mice, which has been similarly reported in mice with AR or other heart diseases (18, 19), we used E/E’ to assess diastolic function as described previously (20). Meanwhile, Tei index was calculated by the following formula: Tei index = (IVRT + IVCT)/ET, as we previously reported (18). To confirm successful establishment of TAC and AR, the peak velocities of aortic arch flow in systole (PSVa) and diastole (PDVa) were recorded, respectively.

**TABLE 1:** Conventional echocardiographic characteristics of mice subjected to pressure or volume overload.

Parameter	Sham-T (*n* = 8)	TAC (*n* = 7)	*P*-value	Sham-A (*n* = 8)	AR (*n* = 9)	*P*-value
HR (bpm)	542 ± 4	532 ± 6	*0*.*07449*	551 ± 13	547 ± 13	*0*.*8057*
PSVa (mm/s)	911.73 ± 67.99	3785.05 ± 227.84**	**1.098E-13	970.44 ± 91.54	1442.65 ± 103.46**	**0.000000126
PDVa (mm/s)	0.00 ± 0.00	0.00 ± 0.00	/	0.00 ± 0.00	427.88 ± 67.52	/
HW/BW	4.68 ± 0.15	6.19 ± 0.04*	*0.0219	4.37 ± 0.06	6.54 ± 0.15*	*0.012
EF (%)	60.05 ± 1.76	58.08 ± 1.77	*0.371*	61.99 ± 1.51	59.58 ± 1.02	*0.0667*
FS (%)	32.77 ± 2.64	31.68 ± 1.16	*0.384*	33.17 ± 1.03	31.79 ± 0.72	*0.141*
LVIDd (mm)	4.11 ± 0.12	4.17 ± 0.23	*0.719*	4.11 ± 0.15	4.99 ± 0.25**##	**0.000172 ##*0.000418*
LVIDs (mm)	2.81 ± 0.23	3.28 ± 0.28	*0.078*	2.81 ± 0.17	4.03 ± 0.17**##	**0.00000371 *##0.00158*
LVPWd (mm)	0.71 ± 0.04	1.05 ± 0.12**	**0.0000571	0.74 ± 0.02	0.81 ± 0.08##	*##0.00157*
LVPWs (mm)	1.09 ± 0.13	1.31 ± 0.06*	*0.0495	1.14 ± 0.08	1.01 ± 0.05##	*##0.00321*
LVEDV (μl)	72.00 ± 2.38	76.66 ± 7.59	*0.487*	75.53 ± 1.67	111.25 ± 5.44**##	**0.00000786 *##0.000306*
LVESV (μl)	23.60 ± 1.30	46.95 ± 3.30**	**0.00481	30.22 ± 1.05	75.00 ± 7.02**##	**0.00000913 *##0.000413*
E/E’	26.66 ± 1.58	39.66 ± 2.76**	**0.000404	27.56 ± 1.47	35.34 ± 5.62**	**0.00471
IVRT (ms)	14.10 ± 0.78	13.26 ± 1.70*	*0.0206	14.37 ± 1.03	15.54 ± 1.48##	*##0.00264*
IVCT (ms)	10.00 ± 1.66	11.58 ± 0.54	*0.0698*	10.62 ± 1.23	15.58 ± 0.96**##	**0.0000590 *##0.00125*
ET (ms)	49.72 ± 3.98	51.67 ± 4.41	*0.826*	51.66 ± 2.96	54.17 ± 2.57##	*##0.0067*
Tei index	0.50 ± 0.14	0.47 ± 0.01	*0.681*	0.56 ± 0.13	0.60 ± 0.04##	*##0.000244*

HR, heart rate; PSVa and PDVa, peak velocities of aortic arch flow in systole and diastole; HW/BW, heart weight/body rate; EF, ejection fraction; FS, fractional shortening; LVIDd and LVIDs, left ventricular internal dimension in diastole and systole; LVPWd and LVPWs, left ventricular posterior wall thickness in diastole and systole; LVEDV and LVESV, left ventricular end-diastolic and end-systolic volume; E/E’, ratio of mitral valve early diastolic maximum velocity to peak mitral annular velocity during early filling; IVRT, isovolumic relaxation time; IVCT, isovolumic contraction time; ET, ejection time. All values are represented as Mean ± SD.

*P < 0.05 vs. corresponding Sham; **P < 0.01 vs. corresponding Sham; ^##^*P* < 0.01 vs. TAC. Italic values represent the statistical values.

### 2.3. Speckle tracking based strain imaging

**FIGURE 2:**
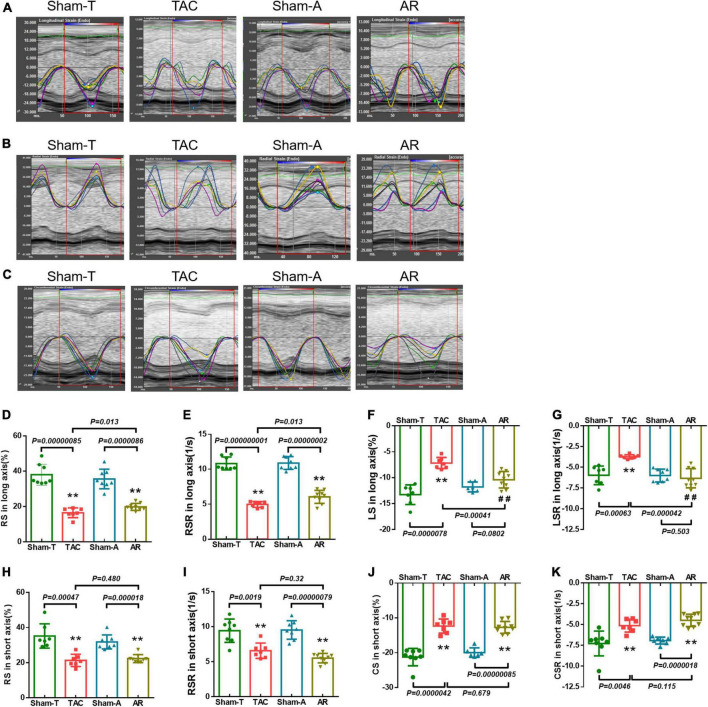
Strain and strain rate in pressure overload and volume overload models. **(A–C)** Representative longitudinal, radial and circumferential strain curves, and time-to-peak analysis in different models. **(D,E)** Global radial strain (GRS) and strain rate (GRSR) in long axis; **(F,G)** global longitudinal strain (GLS) and strain rate (GLSR) in long axis; **(H,I)** GRS and GRSR in short axis; **(J,K)** global circumferential strain (GCS) and strain rate (GCSR) in short axis. Data are shown as mean ± SD. **P < 0.01, compared with corresponding sham-operated group. ^##^*P* < 0.01, compared with TAC group.

**FIGURE 3:**
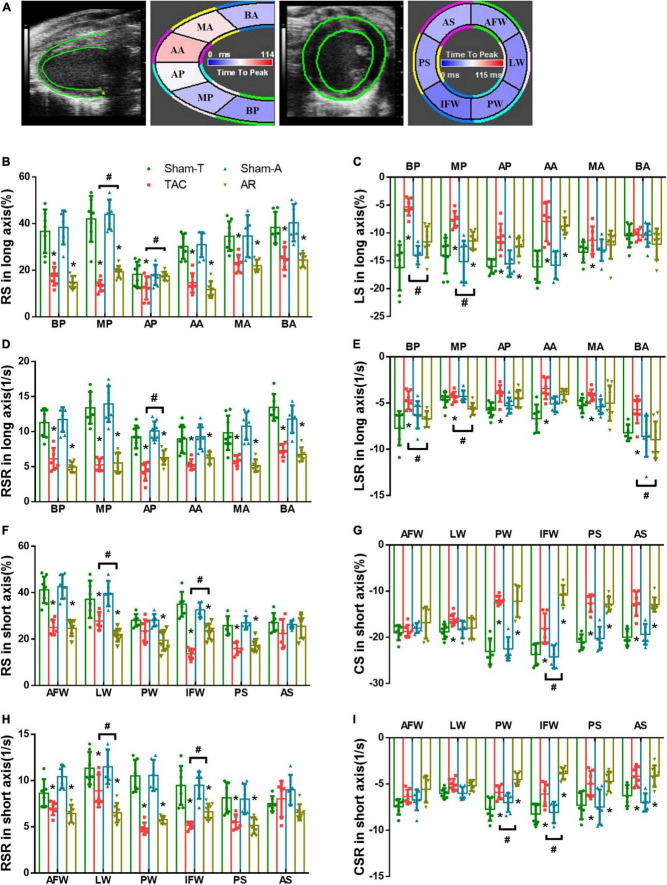
Comparison of changes in regional strain in pressure overload and volume overload mice. **(A)** Tracing the border of endocardium and epicardium and six segments of myocardium in both the long and short axis; BA, basal anterior; MA, mid anterior; AA, apical anterior; AP, apical posterior; MP, mid posterior; BP, basal posterior; AS, anterior septum; PS, posterior septum; IFW, inferior free wall; PW, posterior wall; LW, lateral wall; AFW, anterior free wall; **(B,C)** RS and LS in long axis; **(D,E)** RSR and LSR in long axis; **(F,G)** RS and CS in short axis; **(H,I)** RSR and CSR in short axis; BP, basal posterior; MP, mid posterior; AP, apical posterior; BA, basal anterior; MA, mid anterior; AA, apical anterior; AFW, anterior free wall; LW, lateral wall; PW, posterior wall; IFW, inferior free wall; PS, posterior septum; AS, anterior septum. Data are shown as Mean ± SD. *P < 0.05, compared with corresponding sham group. ^#^*P* < 0.05, compared with TAC group.

The strain imaging analysis was performed using VevoStrain software in an offline workstation (VisualSonics Inc., Toronto, Canada) on the aforementioned B-mode image cineloop acquired at a frame rate higher than 200 fps. Global and regional strain and strain rate were calculated by tracking the movement of endocardium and epicardium border in three consecutive cardiac cycles. Global longitudinal strain (GLS), radial strain (GRS), circumferential strain (GCS), longitudinal strain rate (GLSR), radial strain rate (GRSR), and circumferential strain rate (GCSR) were calculated (Figure 2). In addition to global measurements, regional myocardial strain was available for the six wall segments of the LV automatically assigned by the computer either in the long or short axis view (Figure 3). In the long axis, LV wall was divided into basal posterior (BP), mid posterior (MP), apical posterior (AP), apical anterior (AA), mid anterior (MA), and basal anterior (BA). In the short axis view at mid-papillary level, LV wall was divided into anterior free wall (AFW), lateral wall (LW), posterior wall (PW), inferior free wall (IFW), posterior septum (PS), and anterior septum (AS). All measurements were repeated three times and data processing was performed by a blinded investigator.

### 2.4. Intra and inter-observer variability

The original investigator re-analyzed the echocardiography data at an interval of two weeks to assess the intra-observer variability. Two different observers analyzed the same data to determine the inter-observer variability. Data are presented as means of the absolute differences between measurements and by the interclass correlation coefficient (ICC).

### 2.5. Statistics

All data are presented as means ± standard deviation. All statistical results were obtained using GraphPad Prism v6 (GraphPad Software, San Diego, CA, USA). The continuous variable between two groups were compared by Student’s *t*-test. Multiple comparisons were conducted by one-way analysis of variance (ANOVA) with the Student-Newman-Keuls (S-N-K) test. The conventional echocardiographic assessment had good inter- and intraobserver agreement, as we reported previously (2, 4, 18). Inter- and intraobserver variability of strain related parameters was assessed by ICC using a two-way mixed model with absolute agreement. *P* < 0.05 was considered statistically significant.

## 3. Results

### 3.1. Conventional echocardiography demonstrates preserved systolic function but impaired diastolic function in pressure overload and volume overload mice

Conventional echocardiography was performed to recapitulate the structural and functional changes in TAC and AR murine hearts. Two weeks post-operation, PSVa or PDVa was satisfactorily elevated in TAC or AR mice, respectively (Table 1), indicating pressure overload or volume overload model was successfully established. The heart weight/body weight ratio (HW/BW) was comparably increased in TAC and AR mice. In TAC group, LVPW was remarkably increased compared to Sham-T animals, whereas LVID showed no significant changes, indicating concentric cardiac hypertrophy (Table 1). On the contrary, in AR group, LVPW was comparable to Sham-A counterparts, while LVID was robustly enlarged, suggesting eccentric cardiac hypertrophy (Table 1).

Compared with their corresponding sham mice, both TAC and AR mice showed preserved systolic function as evidenced by comparable LVEF and LVFS. As a sensitive marker for cardiac diastolic function, E/E’ were prolonged in both TAC and AR mice compared to corresponding sham group. As for other parameters of diastolic function, IVRT was decreased in TAC group, whereas it showed a trend of increase but without statistical significance in AR mice. Intriguingly, IVCT was more increased than ET in AR versus TAC mice, leading to an increase in Tei index in AR mice.

### 3.2. Global strain analysis shows more compromised function in TAC mice compared with AR mice

To analyze both inter-observer and test–retest intra-observer reliability of strain related parameters, ICC were calculated and demonstrated in Table 2. All ICC coefficients were greater than 0.80, indicating a satisfactory inter- and intra-observer test–retest reliability.

**TABLE 2:** Intra- and interobserver variability of strain parameters.

	Longitudinal strain	Radial strain	Circumferential strain
Mean	−15.86 ± 8.21	−33.45 ± 8.74	−20.55 ± 2.46
**Intraobserver variability**			
Mean absolute difference, %	5.93 ± 4.58	3.23 ± 2.21	2.25 ± 1.21
ICC	0.97	0.95	0.94
**Interobserver variability**			
Mean absolute difference, %	8.46 ± 3.66	5.52 ± 2.80	2.39 ± 2.28
ICC	0.88	0.93	0.97

Values are means ± SE. ICC, interclass correlation coefficient.

The LV undergoes a complex pattern of deformation which can be examined in the longitudinal, radial, and circumferential directions in both systole and diastole (15). Strain imaging analyses trace the endocardium and epicardium frame-to-frame during the cine loop of cardiac cycles, providing assessment of the global and regional deformation reflected by strain and strain rate in each of these directions. Representative longitudinal, radial and circumferential curves from TAC and AR mice are shown in Figures 2A–C. Analysis was conducted to differentiate global myocardial deformation between TAC and AR mice. In TAC animals, strain measurements revealed that GRS and GLS obtained from parasternal long axis were significantly reduced relative to the corresponding parameters in Sham-T mice. However, in AR group, only GRS from parasternal long axis was decreased versus sham-A animals, while GLS showed no significant differences between AR and sham-operated animals (Figures 2D, F). In addition, GRS and GCS in short axis were significantly reduced in both TAC and AR group (Figures 2H, J). The GRSR, GLSR, and GCSR showed similar results corresponding to their specific global strain measures in the two models (Figures 2E, G, I, K).

### 3.3. Regional strain analysis unveils different pattern of myocardial dysfunction between pressure overload and volume overload models

Strain imaging allows the assessment of regional myocardial function by dividing the LV into six distinct segments in the short- and long-axis (Figure 3A). In order to further investigate the nuanced differences in LV dysfunction caused by TAC and AR, strain and strain rate of each segment were analyzed. Consistent with the results of global strain analysis, RS and RSR of all segments in the long axis were decreased in both TAC and AR groups, whereas the former owned more reduction in MP and AP (Figures 3B, D). Moreover, compared with corresponding sham-operated groups, decreased LS was observed in all segments except BA region in both TAC and AR mice, and the decrease was more pronounced in TAC mice. LSR decreased greatly in BP, MP, and BA region only in TAC group, while no significant alterations were detected in AR group, although LSR showed a declining trend with no statistical significance (Figures 3C, E).

Consistently, in both TAC and AR mice, regional strain analysis in short axis also revealed decreased RS and RSR in most segments, with the exception of AS segment, in which RS and RSR were comparable among all groups. (Figures 3F, H). CS and CSR of the two group were significantly decreased in PW, IFW, PS, and AS (Figures 3G, I). Specifically, CS and CSR changes in PW and IFW segments were slightly more dented in AR model than TAC model (Figures 3G, I). CS and CSR were largely preserved in AFW and LW in both murine models (Figures 3G, I).

### 3.4. Observer variability data

Intra- and interobserver variability of speckle tracing data are analyzed and shown in Table 2. In general, these results revealed acceptable intra- and interobserver variabilities.

## 4. Discussion

In this study, speckle tracking based strain imaging was employed to comparatively assess the cardiac function in mouse models of pressure overload and volume overload. Our results revealed that in compensated cardiac remodeling reflected by preserved LVEF and LVFS, strain imaging could detect early subtle changes in myocardial deformation and cardiac function, and LS and LSR could be used to distinguish functional changes in myocardial hypertrophy induced by different mechanical overload, being more sensitive than the conventional echocardiographic measurements.

Although conventional echocardiography parameters, such as LVEF, LVFS, and E/E’, are widely used in assessing cardiac structure and function in clinical and basic studies, it reflects the overall performance of heart and lacks sufficient sensitivity to reflect subtle cardiac function (10, 15, 21). Thus, changes detected by conventional echocardiography are often considered late manifestations of disease (15), making it difficult to identify early changes in cardiac function of the two types of hypertrophic hearts, for both pressure overload and volume overload induced cardiac remodeling characterized by myocyte enlargement and loss, fibrosis, metabolic abnormalities, and mitochondrial dysfunction, in “compensated” cardiac hypertrophy (22). Nevertheless, in clinical practice, subtle abnormalities in myocardial activity are typically uncovered by strain imaging even in the setting of normal cardiac function by conventional measures (21). Myocardial strain measurement was associated with subclinical ventricular dysfunction and the outcome in patients with aortic stenosis and preserved LVEF (22, 23). Moreover, strain analysis was applied to assess the left ventricular contraction patterns and the severity of cardiac remodeling in patients with chronic aortic regurgitation and preserved ejection fraction (24, 25). In basic studies, recently developed strain imaging designed specifically for rodents allows for early detection of intrinsic myocardial dysfunction in the setting of pressure overload, myocardial infarction, aging, and epirubicin treatment (6, 10, 15, 26). We in this study further found that when LVEF and LVFS remained normal, the strain and strain rate in radial and circumferential direction were decreased in both TAC and AR mice, suggestive of higher sensitivity and superiority of strain imaging over conventional echocardiography in murine models of mechanical stress.

Strain analysis is based on combined speckle tracking algorithms, thus strain and strain rate are supposed to reflect the global and regional myocardial function (14, 27). In this study, we found that LS and LSR were decreased evidently in TAC mice but largely unchanged in AR mice. Our findings suggest that LS and LSR could be used to distinguish the effects of different types of mechanical overload. It is documented that LS related parameters mainly depend on the condition of sub-endocardial myofibers and thus reflects the subendocardial status (6, 28). Recent studies further reported that LS is a particularly sensitive marker of the subendocardial myofiber dysfunction that occurs early during hypoperfusion or mechanical stress (15, 29). Coincidingly, previous studies from us and others indicate lateral hypertrophy in TAC cardiomyocytes while longitudinal hypertrophy in AR cardiomyocytes (3, 18). Thus, the stimulus-specific remodeling pattern of cardiomyocytes may contribute to the difference of LS related parameters in these two models.

Myocardial fibrosis defined as excessive deposition of extracellular matrix proteins, including collagens, is another conspicuous characteristic in the hearts of mechanical overload (30). Fibrosis increases ventricular stiffness and diffusion distance of oxygen to cardiomyocytes, leading to impaired cardiac function (3, 30). Previous comparative studies, including ours, demonstrate more prominent cardiac fibrosis is in TAC versus AR hearts (3, 4, 18). Based on these, we speculated that the distinguishing effect of LS and LSR may be closely associated with differential fibrosis between pressure and volume overload hearts. In addition, Bi and colleagues recently demonstrated in epirubicin treated mice that the collagen content in sub-endocardial layer was more prominent and LS was more reduced compared with that in sub-epicardial layer (6), strengthening the notion that LS and LSR served as the most specific indicator of all strain indexes.

Pressure overload and volume overload induce concentric and eccentric hypertrophy, respectively (4). The two types of cardiac hypertrophy also manifest different sensitivity and outcomes to similar therapeutics (3, 18). It is still controversial that which type is more maladaptive than the other. Several studies have suggested that volume overload is more harmful to the ventricle, because an augmentation of end-diastolic dimension and wall stress is already presented in the compensated phase of aortic regurgitation (21). On the other hand, other studies, including one from ours, have proposed that pressure overload is more detrimental, as pressure overload causes more fibrosis. The more prominent fibrosis in TAC mice is supposed to be associated with more activation of pro-hypertrophy effectors including Ca^2+^/calmodulin-dependent protein kinase II (CaMKII) (4), which induces NLRP3 inflammasome activation in response to pressure overload (31). The novelty of this study is to investigate this issue by a direct comparison using state-of-the-art strain imaging, and unveil that LS and LSR are reduced in pressure rather than volume overload cardiac hypertrophy. Our findings thus support the notion that volume overload induces a more benign phenotype of cardiac hypertrophy.

### 4.1. Limitations

A comparative exploration in the association between cardiac fibrosis and strain alteration would more comprehensively elucidate the role of fibrosis in the discrepancy of strain changes between TAC and AR hearts. However, our previous study showed that cardiac fibrosis was more prominent in TAC heart and suggested that fibrosis contributed to the discrepancy. More in-depth analyses in different sections (such as sub-endocardial layer versus sub-epicardial layer) are warranted in future studies.

## 5. Conclusion

By using high frequency ultrasound, we successfully undertake global and regional strain analyses in mouse models of pressure overload and volume overload cardiac hypertrophy, and discover that reduction of strain and strain rate precedes changes of conventional echocardiographic parameters such as LVEF and LVFS. Pressure overload causes more reduction of strain and strain rate than volume overload, identifying LS and LSR as the most specific indicator of all strain indexes to differentiate left ventricular functional changes under distinct mechanical overload. The stimulus-specific heterogeneity in strain measurements makes it a sensitive approach for early detection of myocardial dysfunction in mechanistic and pharmacological studies.

## Data availability statement

The original contributions presented in this study are included in this article/supplementary material, further inquiries can be directed to the corresponding authors.

## Ethics statement

The animal study protocol was approved by Animal Care and Use Committee of Zhongshan Hospital, Fudan University and were in accordance with National Institutes of Health Guide for the Care and Use of Laboratory Animals (No. 85-23, revised 1996, Bethesda, MD, USA).

## Author contributions

JW, YZ, and RX designed the study. RX, ZD, and JW predominantly performed echocardiography and data collection. RX, ZD, HL, JS, and LC analyzed the data. RX and ZD wrote the manuscript. HX, JW, and YZ revised the manuscript and provided critical advice. All authors read and approved the final version of the manuscript.
